# Induced Pluripotent Stem Cells and Outer Retinal Disease

**DOI:** 10.1155/2016/2850873

**Published:** 2016-01-05

**Authors:** Jin Yang, Bingcui Cai, Patrick Glencer, Zhiqing Li, Xiaomin Zhang, Xiaorong Li

**Affiliations:** ^1^Tianjin Medical University Eye Hospital, Tianjin 300384, China; ^2^Nova Southeastern College of Optometry, Fort Lauderdale, FL 33314, USA

## Abstract

The retina, which is composed of multiple layers of differing cell types, has been considered the first choice for gene therapy, disease modeling, and stem cell-derived retinal cell transplant therapy. Because of its special characteristics, the retina, located in the posterior part of the eye, can be well observed directly after gene therapy or transplantation. The blood-retinal barrier is part of a specialized ocular microenvironment that is immune privileged. This protects transplanted cells and tissue. Having two eyes makes perfect natural control possible after a single eye receives gene or stem cell therapy. For this reason, research about exploring retinal diseases' underlying molecular mechanisms and potential therapeutic approach using stem cell technique has been developing rapidly. This review is to present an up-to-date summary of the iPSC's sources, variations, differentiation methods, and the wide-ranging application of iPSCs-RPCS or iPSCs-RPE on retinal disease modeling, diagnostics, and therapeutics.

## 1. Induced Pluripotent Stem Cells

Stem cells are characterized by their ability to divide and differentiate into specialized cell types and by their capacity to self-renew to produce more of the same type of cell. In the past, embryonic stem cells (ESCs), with their ability of unlimited proliferation and somatic cell differentiation, had been considered as the source of regenerative medicine. Ethical issues and lifelong immune rejection limited the modeling and transplant therapy in the clinical setting. Recent breakthroughs occurred in reprogramming stem cells directly from adult somatic cells, bypassing the need for embryonic stem cells. In 2006, it was shown that transducing cells with a series of four transcription factors (Oct4, Sox2, Klf4, and c-Myc) into somatic cells enabled reprogramming DNA into “stem cells” [1]. The resultant pluripotent stem cells, or induced pluripotent stem cells (iPSCs), coming from somatic cells, have personal genetic or protein information that may have the potential for personalized therapeutic approaches. Some aging diseases, including AMD, have been reported to be related to multiple haplotypes and have developed disease by reacting with environmental risk factors making it difficult to model; however, they can be modeled on a dish by culturing personalized iPSC-derived retinal cells. Patient-derived stem cells can also sidestep the problems of immune rejection and the ethical issues associated with ESCs.

### 1.1. Sources of iPSCs Cells

Since iPSCs can come from the patient's somatic cells, various somatic tissues have been tried as sources of iPSCs [2–6]. Many of these experiments tested genetic labeling or gene expression, competent enough to generate germline chimeras or other techniques to confirm the identity of iPSCs with the characteristics of embryonic stem cell. Skin cells were still the most commonly used and predominant source of iPSCs before more noninvasive methods have been developed.

The sampling of somatic cells is invasive. The difficulty of sampling stomach cells, liver cells, and so forth has limited their application, limiting the recruitment of large numbers of potential donors. A lesser invasive or noninvasive detection requirement is making the search for optimal, reliable, and safe sources for iPSCs reprogramming continue. Blood is considered an ideal source of cells for reprogramming because of its abundance and accessibility [7]. Blood from bone marrow and cord blood had been considered a reliable source at the beginning [8, 9]. Peripheral blood reprogramming techniques using T cells and red cells have been developed [10, 11]; 2–6 mL of peripheral blood can purify enough CD34+ cells for reprogramming. Until recently, finger-prick-derived iPSCs were generated from different donors at very high efficiency (100–600 colonies per milliliter of blood) as long as 20,000–30,000 cells can be collected [12], making reprogramming possible during routine physical test procedures. Noninvasive sampling could make it much easier to recruit people for donation. Urine and hair are considered the most acceptable sampling sites [13, 14]. Dr. Xue et al. described a practical method to generate human iPS cells from urine-derived cells (UCs) under feeder-free, virus-free, and serum-free conditions and without oncogene c-Myc [13], while enough epithelium cells have to be collected from the urine; hair follicle dermal papilla (DP) cells cultured in a medium supplemented with valproic acid at a physiological level of oxygen (5%) increased the efficiency of DP cells reprogramming in dermal fibroblast from 0.01% to 0.03% [14].

Whether the origin of the parental cell ultimately determines the behavior of the resultant iPSCs cell line is an active debate. Hu et al. hypothesized that reprogrammed cells retain a “memory” of their origin in terms of propensity for differentiation [15]. They reprogrammed primary fetal RPE cells first to iPSCs. After the removal of FGF2, the cells spontaneously differentiated back into RPE, showing the possibility of reprogrammed cells tending to “dedifferentiate” into their former identity. Chromosome microduplication in somatic cells can decrease the genetic stability of human reprogrammed somatic cells, showing that the behavior of resultant iPS cell lines can be affected by the state of the original cells [16]. This phenomenon of a resultant cell line still keeping remnants of epigenomes and transcriptomes of the donor tissue has been discussed and summarized [17–19]. These residual signatures of epigenomes and transcriptomes of the somatic tissue of origin were termed “epigenetic memory.” Thus, it would be reasonable to use the biopsy material of cells of the same origin if possible [20].

### 1.2. Differentiation of iPSCs to Outer Retina

Generation of iPS-derived retinal cells and tissues from individuals with retinal disease is a rapidly developing technology that holds the possibility for the autologous transplantation and disease modeling.

Retinal pigment epithelium (RPE) is a highly pigmented monolayer of cells that is located between the retinal photoreceptors (PR) and choriocapillaries with high polarization. The basal side of the cells attaches to the basement membrane while the apical side contains microvilli and faces the photoreceptor segment tips; this is critical to maintain PR function. Primarily, RPE dysfunction may initiate PR atrophy and choriocapillary loss leading to AMD or Retinitis Pigmentosa. Since the RPE cells are the same sources of neural ectoderm, this makes spontaneous differentiation of iPSCs into RPE possible [21]. With bFGF deprivation [22], RPE-like cells can be derived using various iPSCs cultured media after 10–12 weeks of differentiation, which shows a large number of pigmented colonies of hexagonal RPE-like cells expressing the RPE-specific genes. They are then needed to be manually microdissected to generate an enriched culture of RPE. There are other methods that can be used to get RPE cells from iPSCs: Nicotinamide (NIC), with or without Activin A, treats cells [23] and was called forced induction of iPS/hESC to RPE [24]. This can help direct differentiation of optic vesicles to RPE and increase pigmented cells, making differentiation times a lot shorter. Recently, rapid differentiation of RPE cells from iPS cells has been developed. They lasted 14 days by only using retinal inducing factors (IGF1, Noggin, Dkk1, and bFGF) and other factors (NIC, Activin A, SU5402, and vasoactive intestinal peptide (VIP)) [21]. However, the expensive conditions of this experiment may limit its wide-range use.

Differentiation into photoreceptors under specifically defined culture conditions from ES and iPSCs has been shown in several papers. It is more complicated than RPE differentiation. First of all, extrinsic chemical factors are needed to modulate specific signaling pathways. Combination of Wnt, Nodal, and Notch pathway inhibitors (Noggin, Dk1, LeftyA, and DAPT) and other growth factors can help retinal progenitor cells form [25, 26]. Further differentiation into photoreceptors has been tried on 2D culture systems. They additionally require exposure to native retinal cells in coculture systems, RX+ or Mitf+ by subsequent treatment with retinoic acid and taurine [26, 27], or to several exogenous factors including Noggin, Dkk1, DAPT, and insulin-like growth factor [28]. Because it is hard to get photoreceptor-light response after transplantation by photoreceptors under 2D cultures systems, inducing iPS cells into functional mature photoreceptors and integrating with RPE cells have been tried numerous times. For the functional integration, transplants with rod photoreceptor precursors differentiate up to 28 days by avoiding the early stage (producing large tumors). Prolonged differentiation can show better integration capacity [29]. Forming optic vesicle-like structures in 3D culture systems, with each aggregate possessing the ability to differentiate into all major retinal cell types, allows for photoreceptors to integrate and functionally mature much easier [30, 31]. Meyer et al. reported the ability of select human ES and iPS cell lines to differentiate into retinal progenitor cells without the need of exogenous factors [31]. 200 days of photoreceptor differentiation can culture 3D retinal tissue that can generate all major retinal cell types including neurons (ganglion, amacrine, horizontal, bipolar, rod, and the three types of cones) and Müller glial cells, all arranged in their proper layers [32]. Zhong et al. speculate that physical microenvironmental cues (especially cell-cell and cell-extracellular matrix interactions) at the initial stages of iPSC differentiation are key to the establishment of this retina differentiation niche.

## 2. Using iPSCs to Model Outer Retina Disease

Many different forms of blindness result from the dysfunction or loss of the outer retina. Hereditary retinal degeneration with various different gene mutations is increasingly becoming the leading course of irreversible blindness in Western nations [33]. Age-related macular degeneration (AMD) is the leading cause of blindness among the elderly in developed countries [34]. Both knowing the mechanisms and the way to apply therapy to these outer retinal diseases pose a significant challenge. iPSCs derived from somatic cells of patients can be differentiated to different retinal layers stepping closer to the anticipated use of iPSCs for disease modeling and future therapies [35].

### 2.1. Retinitis Pigmentosa (RP)

RP is characterized by progressive loss of rod photoreceptors or RPE cells. Symptoms of RP include night blindness and progressive visual field loss that can often lead to complete blindness. With progress in the field of molecular genetics, genetic factors are known to play a significant role in the pathogenesis of multiple retinal degeneration cases. Over 60 different genes have been associated with RP. Described inheritance patters include autosomal dominant (15–35%), autosomal recessive (60%), X-linked (5–18%), and mitochondrial. A number of “disease-in-a-dish” iPSC models have been engineered for identifying possible pathogenicity and therapeutic strategies.

Patient specific photoreceptors differentiation from the patients with RP1, RP9, RPPH2, or RHO gene mutation and drug testing on these cells have been reported by Jin et al. [36]. Cells derived from patients with a specific mutation expressed markers for oxidation or endoplasmic reticulum stress and exhibited different responses to vitamin E from what had been observed in clinical processes. The antioxidant vitamins *α*-tocopherol, ascorbic acid, and *β*-carotene have been tested directly on patient iPS cell-derived photoreceptors. The results showed that *α*-tocopherol had treatment effects in iPS cells derived from RP9 mutation helping to narrow down the disease targets for experimental drugs.

Confirming and studying a patient's specific disease-causing mutations by using iPSCs technique can explore the underlying molecular mechanisms. The fact of E181K rhodopsin mutation, which was correlated with the increased expression of endoplasmic reticulum (ER) stress and apoptotic markers leading to rod photoreceptor death, was confirmed by introducing the mutation into wild-type control iPSCs. It was amended by rescuing patient-derived cells [37]. The pathogenic mechanisms of Membrane Frizzled-Related Protein (MFRP) mutation were unknown. Li and colleagues used human iPSC-RPE cells and elucidated that MFRP could control actin organization with the help of CTRP5 [38]. Gene therapy approach also showed MFRP-associated RPE successfully restored actin organization in this paper. Usher syndrome is an autosomal recessive hereditary disorder with RP and congenital sensorineural hearing loss phenotype. Multilayer eyecup-like structures with features of human retinal precursor cells derived iPSCs from the USH2A gene mutated patient's keratinocytes have been differentiated by Tucker et al. [39]. Analysis of the USH2A transcripts of these cells revealed that USH2A gene contained an unspliced intronic sequence that causes a premature stop codon. Protein expression revealed upregulation of GRP78 and GRP94, suggesting that the patient's other USH2A variant (Arg4192His) causes disease through protein misfolding and ER stress. Leber's congenital amaurosis (LCA), also known as early-onset retinal dystrophy, had several patients who were blind from birth but born to normal-sighted parents. LCA is characterized by poor fixation in the first months of life coinciding with sensory nystagmus and amaurotic or sluggish pupils. CEP290 gene causes one-third of patients to have LCA, a gene that produces a cilium-associated protein [40]. Burnight and colleagues [41] developed patient-specific, iPSC-derived, photoreceptor precursor cells. The disease-specific phenotype of CEP290-associated LCA patients to form cilia was investigated. They found that the cilia that were formed were shorter in patient-derived cells than in cells from unaffected individuals. They also made lentiviral delivery of CEP290 rescue the ciliogenesis defect successfully.

### 2.2. AMD

Age-related macular degeneration (AMD) is one of the leading causes of severe vision loss in individuals over the age of 55 [34]. Despite intensive basic and clinical research, its pathogenesis remains unclear. Studies have shown that both genetic factors and environmental factors are involved in the onset of AMD. 50 different loci have been identified in AMD patients while chromosomes 1 and 10 lead to a high-risk haplotype. Because these haplotypes are known to be a risk factor for AMD, Yang et al. created specifically genotyped AMD disease models using iPSC technology for use as valuable tools to find out more about AMD's molecular pathways [42]. 10 *μ*M A2E and blue light artificially instill an aging process used in iPSC-derived cell lines recapitulating the phenotype of a late-onset degenerative disease, which provided an ideal system for modeling AMD and highlighted lower levels SOD2 activity in high-risk alleles that may be one of the underlying pathogenic mechanisms. Best's disease (BD), another inherited degenerative disease of the human macula, has the phenotype of accumulation of subretinal fluid and autofluorescent waste products from shed photoreceptor outer segments (POSs). Singh and colleagues [43] used POS feeding RPE from mutant iPSCs that showed disrupted fluid flux and increased accrual of autofluorescent material after long-term exposure when compared with iPSC-RPE from unaffected siblings; this can be treated as another aging system for RPE cells. Chang et al. treated iPS-RPE cells which were derived from dry AMD patients with 10 *μ*M curcumin and showed a significant effect on cell viability [44]. Most recently, Singh group continued their research by applying valproic acid (VPA), with or without rapamycin; this increased rates of POS degradation in iPS-RPE model, whereas application of bafilomycin-A1 decreased such rates [45]. Their findings may contribute to the possibility of BD or other macular diseases that can be manipulated pharmacologically. Modeling these diseases, including both genetic and environmental factors, by using iPS-RPE, is very useful in understanding the pathogenesis and the development of effective therapeutic strategies.

## 3. RPE and Photoreceptor Transplantation

Over the past decade, RPE or photoreceptor progenitor cell transplantation from ESC as a means of replacing tissue has evolved rapidly. Recently, because of the obstacle of immune rejection, iPSCs derived retinal cells have a more promising future.

Since the RPE cells from a patient's own cells can have a better ability to interdigitate with the photoreceptor outer segments and support them, two different therapeutic strategies have been tried to optimize RPE transplantation procedures: subretinal injection of the cell suspension of nonpolarized iPS-RPE cells and transplanting polarized RPE monolayer sheets. Because normal RPE physiology needs a polarized monolayer of tight junctions, post-cell suspension transplantation of the monolayer formation is the most important part for successful transplantation. While recent studies showed that the suspended cells preferentially accumulated at the lower margin of the subretinal injection bleb 7 days after injection [46], there is the possibility of formation of multilayered clumps of cells or tumors that could damage the retina. Therefore, RPE sheet transplantation is considered a better alternative. The key to produce a natural anatomy of a polarized monolayer's RPE for transplantation is the carrier. A carrier substrate that mimics Bruch's membrane support of the monolayer of RPE cells will allow better controlled surgical delivery into the subretinal space. Nonbiodegradable substrates like Parylene [47], poly scaffold [48], and cross-linked gelatin scaffold [49] have been tried as RPE carriers because of their potential advantages. They have good mechanical strength and biostability, support RPE growth and polarization, and so forth. While using biodegradable substrates is proposed as a better means for delivering RPCs to the subretinal space [50, 51], the degradable material effect on peripheral tissue should be considered. Kamao et al. used type 1 collagen gel as a base for cells seeded to form iPS-RPE monolayer. After the monolayer formed, collagenase can be used to dissolve the base before transplant. The whole sheets can be floated up for transplant without a carrier [46].

As stated before, iPSCs can differentiate to progenitor photoreceptors, 3D retina. The progenitor photoreceptors combined with RPE graft or 3D retina-RPE tissue should all be tried on different animal models. The safety and functional recovery are the two most important parts to study. Undifferentiated pluripotent stem cells can differentiate into all cell types of the three germ layers, which is called tumorigenesis. Preclinical animal studies are needed to exclude the possibility of tumorigenesis before a clinical trial should take place. Advances in clinical imaging and functional assessment technique, such as OCT and Electroretinography, can be very useful in animal studies. OCTs can show different retinal layers on vital animals and Electroretinography envisions electrical responses of the retina [52]. For clinical trials, good manufacturing practices (GMP) should be enforced and monitored as early as the differentiation stage. There are at least 14 ongoing clinical trials that are summarized by Nazari et al. [24]. There was only one clinical trial related to iPSCs derived cells used for human RPE cells replacement. Japan's Ministry of Health approved the first clinical study using RPE derived from iPSCs for the treatment of AMD. A 70-year-old female with AMD received 1.3 × 3 mm RPE sheet transplantation. This clinical trial was held by Takahashi et al. First, the CNV was removed followed by iPS-RPE sheet transplantation in this patient (2015 Asian-ARVO). The standardization of this operation may help these clinical treatments go from academic research towards commercialization of iPS-RPE cells transplantation therapy. The following is a big question that should be asked: when should the transplant procedure be considered? This is controversial because the RPE and the photoreceptor cells become atrophied at the more advanced stages of AMD. Transplant of the RPE by itself cannot save the visual acuity. Early treatments are still debatable considering that vision regeneration is quite slow in AMD patients, and combined transplantation of photoreceptor and RPE cells is not yet proven to be technically safe or efficient enough for clinical therapy [24]. Another challenge is that the personalized therapeutic concept of iPSCs is making it hard to progress toward commercialization. Pursuing comprehensive iPSC banking should be contemplated. The iPSCs could be classified by different HLA genotypes making the commercialization of iPSCs derived cell therapy possible [53]. The patient could be saved by other people's iPSCs derived with the same HLA genotype as him or herself.

## 4. Conclusion

IPSC's technology is coming of age as a tool to recapture normal versus abnormal retinal cell behavior in vitro and be applied to the mechanisms of different retinal diseases. There are many emerging innovating technologies that can be combined with iPSCs offering an exceptional opportunity to treat inherited retinal degenerative diseases and save the patient's visual functions. The iPSCs derived cell replacement therapies are still under trials to test safety, efficacy, long-term functionality, cost, and technical complexities of the procedures. These issues need to be overcome in the near future. Increased funding opportunities, a high level of commitment for collaboration, and research sharing are needed in the coming years to benefit the patients.
